# Biological Maturity Status in Elite Youth Soccer Players: A Comparison of Pragmatic Diagnostics With Magnetic Resonance Imaging

**DOI:** 10.3389/fspor.2020.587861

**Published:** 2020-12-15

**Authors:** Daniel Leyhr, Dennis Murr, Lajos Basten, Katrin Eichler, Thomas Hauser, Dennis Lüdin, Michael Romann, Giuseppe Sardo, Oliver Höner

**Affiliations:** ^1^Department of Sport Psychology and Research Methods, Institute of Sports Science, Eberhard Karls University of Tübingen, Tübingen, Germany; ^2^Methods Center, Eberhard Karls University of Tübingen, Tübingen, Germany; ^3^Department of Diagnostic and Interventional Radiology, Goethe-University Hospital Frankfurt, Frankfurt, Germany; ^4^Deutscher Fußball-Bund, Frankfurt, Germany; ^5^Swiss Federal Institute of Sport Magglingen SFISM, Department of Elite Sport, Magglingen, Switzerland

**Keywords:** talent development, youth football, talent identification, biological maturation, MRI

## Abstract

The influence of biological maturity status (BMS) on talent identification and development within elite youth soccer is critically debated. During adolescence, maturity-related performance differences within the same age group may cause greater chances of being selected for early maturing players. Therefore, coaches need to consider players' BMS. While standard methods for assessing BMS in adolescents are expensive and time-consuming imaging techniques (i.e., X-ray and MRI), there also exist more pragmatic procedures. This study aimed to evaluate commonly used methods to assess BMS within a highly selected sample of youth soccer players. A total of *N* = 63 elite male soccer players (U12 and U14) within the German Soccer Association's talent promotion program completed a test battery assessing BMS outcomes. Utilizing MRI diagnostics, players' skeletal age (SA_MRI_) was determined by radiologists and served as the reference method. Further commonly used methods included skeletal age measured by an ultrasound device (SA_US_), the maturity offset (MO_MIR_), and the percentage of adult height (PAH_KR_). The relation of these alternative BMS outcomes to SA_MRI_ was examined using different perspectives: performing bivariate correlation analyses (1), modeling BMS as a latent variable (BMS_lat_) based on the multiple alternative diagnostics (2), and investigating individual differences in agreement (3). (1) Correlations of SA_MRI_ and the further BMS variables ranked from *r* = 0.80 to *r* = 0.84 for the total sample and were lower for U12 (0.56 ≤ *r* ≤ 0.66), and U14 (0.61 ≤ *r* ≤ 0.74) (2). The latent structural equation modeling (SEM) (*R*^2^ = 51%) revealed a significant influence on BMS_lat_ for MO_MIR_ (β = 0.51, *p* <0.05). The additional contribution of PAH_KR_ (β = 0.27, *p* = 0.06) and SA_US_ (β = −0.03, *p* = 0.90) was rather small (3). The investigation of individual differences between the reference method and alternative diagnostics indicated a significant bias for MO_MIR_ (*p* <0.01). The results support the use of economical and time-efficient methods for assessing BMS within elite youth soccer. Bivariate correlation analyses as well as the multivariate latent variable approach highlight the measures' usefulness. However, the observed individual level differences for some of the utilized procedures led to the recommendation for practitioners to use at least two alternative assessment methods in order to receive more reliable information about players' BMS within the talent promotion process.

## Introduction

In the context of competitive sports, major sports organizations invest considerable financial resources and work in the promotion and development of youth players (Johnston et al., 2018), meaning a huge commitment for the organizations as well as for the players. For instance, upon being drafted (to a selection squad), promising players are removed from their familiar environment at an early stage (Baker et al., 2018). While this separation can be valuable from a performance-oriented perspective, it can also represent a serious interruption in the personality development of young players (Fraser-Thomas et al., 2008). Therefore, a fair (and optimal) selection process must take into consideration the viewpoints of both competitive sports and pedagogical development. However, several studies point out that such selection processes within talent development programs are challenging in youth soccer (Gouvêa et al., 2017; Cumming, 2018).

A major reason for this problem is separating youth players into chronological age (CA) groups (e.g., U10, U11, and U12) based on an annual cutoff date (Helsen et al., 2005; Deutscher Fußball-Bund, 2020). These classifications lead to CA differences of players within an age group of up to 1 year (Malina et al., 2019). Players born early in a given year (i.e., first birth quarter) generally have a physiological advantage in their development in contrast to their younger counterparts (i.e., those born in the fourth quarter). This leads to the well-known relative age effect (RAE), which occurs especially in soccer talent identification (Deprez et al., 2013). Votteler and Höner (2017) emphasize the importance of this effect by demonstrating that significantly more players are born in the first rather than last quarter in German youth national teams. Moreover, this problem is further reinforced when a player's biological maturity status (BMS), regardless of his CA, is neglected. Johnson et al. (2017) point out that BMS has a stronger impact than RAE when selecting players. Especially in the pubertal stages (i.e., 11–16 years; Deprez et al., 2015), in which rigorous and important selection processes take place, the difference in players' BMS can reach up to 5 years (Malina et al., 2004). In practice, this leads to the phenomenon where coaches and talent scouts often prefer early maturing players due to a currently better performance level based on more developed physical attributes (Unnithan et al., 2012). At the same time, late maturing players show lower performance levels, especially in physiological predictors, and therefore are often overlooked (Cumming et al., 2017). Hence, those players often do not receive access to a comprehensive talent promotion program with more qualified coaches and better resources. Furthermore, they also receive less playing time in competitive matches, less team responsibility, and less emotional support, which undermines their holistic soccer education (Malina et al., 2015). In the worst case, even highly talented players are deselected due to the time-delayed biological development in comparison with their on-time or early developing peers. Therefore, sports scientific research on talent has long shown that a player's individual BMS should be taken into account within talent promotion, particularly in selection processes (Romann and Javet, 2018).

As a result, there have been recent initial approaches that have tested the classification of players according to their biological maturity rather than CA. This classification strategy is currently referred to as bio-banding (Cumming et al., 2017). Bio-banding can be considered in different domains, such as conducting new competition formats and grouping players in strength and conditioning training to prevent injuries or in talent identification with respect to selecting players for promotion programs (see Cumming et al., 2018a,b). To date, only a few pilot studies have evaluated bio-banding, e.g., within tournaments initiated by United States Soccer Federation or as a part of the Elite Player Performance Plan by the English F.A. (Bradley et al., 2019).

These studies indicate that bio-banding offers potential benefits for talent promotion programs (Malina et al., 2019). However, in order to obtain the benefits of implementing bio-banding in soccer practice and research, appropriate methods are needed that meet ethical and economically pragmatic criteria and undergo a sound psychometric evaluation.

To determine BMS in youth players—both in research and in practice—various methods are proposed that can objectively assess different dimensions of biological maturity (e.g., skeletal age or somatic age; Lloyd et al., 2014) or qualitatively through a morphological, subjective examination of the maturity status by experts (Romann et al., 2017). In the research literature, measuring skeletal age radiographically is currently regarded as the gold standard method (Lloyd et al., 2014). In various international talent studies, an image of the left hand is taken by using an X-ray (Gouvêa et al., 2017; Holienka et al., 2017). However, some researchers have figured out that even in different assessment methods based on X-rays (i.e., Fels method vs. Tanner–Whitehouse 3 method; Malina et al., 2007a), no satisfying agreement in the determination of skeletal age could be achieved. Furthermore, due to the radiation exposure, there are ethical concerns that make routine implementation very difficult, especially with youth and adolescent players (Focardi et al., 2014). In fact, in competitive sports in many European countries, there is currently no legal basis for using X-ray examinations to estimate the ages of healthy youth players (Timme et al., 2017). In this context, several studies mention that, in addition to the X-ray method as the gold standard, an image of the hand-wrist can also be reliably produced by radiation-free magnetic resonance imaging (Dvorak et al., 2007; Dvorak, 2009; George et al., 2012; Bolívar et al., 2015; Urschler et al., 2016; MRI). Going further, there is also evidence that MRI that does not use ionizing radiation is fundamentally more accurate than X-rays due to its high contrast resolution (Serinelli et al., 2015). Therefore, currently—from the authors' point of view—MRI is the most established method for determining skeletal age and can be used without ethical consequences.

In general, the MRI method is associated with economic disadvantages (e.g., high costs and long acquisition time) for practical use. Therefore, there appears to be a need for less costly and less technical methods to measure the biological maturity based on skeletal age. One promising alternative method involves a newly developed ultrasound diagnostic device (SonicBone, Rishon Lezion, Israel). Currently, however, this method has been only sufficiently validated for children, but not in a sport-specific context (i.e., Rachmiel et al., 2017; Utczas et al., 2017), and a validation of this method in the field of youth competitive sports is still pending. This validation is necessary given the fact that moderator variables have to be considered in talent research (e.g., performance level, age groups, gender, see Murr et al., 2018).

To date, practitioners from diverse talent promotion programs mainly use alternative assessments and determine biological maturity by somatic age (Cumming et al., 2018b). In this context, two commonly utilized methods are the calculation of the maturity offset (MO) based on the estimation of individual age at peak height velocity (APHV; Mirwald et al., 2002) and calculating the percentage of predicted final adult height (PAH; Khamis and Roche, 1994). More specifically, the Mirwald method estimates the players' MO based on various parameters (CA at the time of measurement, weight, height, sitting height, and leg length), while the Khamis–Roche approach predicts the adult height from weight and height of the individual as well as the height of the biological parents. However, considering the influence of moderator variables, researchers emphasize that these methods have inaccuracies depending on which age group was studied (Myburgh et al., 2019).

Within the constraints of talent promotion programs, empirical knowledge of appropriate diagnostics for determining BMS is needed. However, in practice, such methods have to be both acceptable with regard to costs and ethical issues for bio-banding strategies and scientifically sound in terms of psychometric properties.

Indeed, in prior studies, researchers aimed to validate different diagnostics to assess BMS in youth players in different sports (e.g., Malina et al., 2007 in American football; Malina et al., 2012 and Romann et al., 2017 in soccer; Myburgh et al., 2019 in tennis). Malina et al. (2007b) found a correlation of *r*_*s*_ = 0.52 between skeletal age measured by left hand-wrist radiographs and PAH in their study with 143 male American football players (9.27–14.24 years). By utilizing the same method as Malina et al. (2007b), similar results were detected by Myburgh et al. (2019) in an investigation with 40 male, British junior tennis players (12.5 ± 1.8 years). Apart from PAH, this study also used predicted APHV (Mirwald et al., 2002) for BMS assessment and found lower correlations between various assessment methods (PAH: *r*_*s*_ = 0.35; predicted APHV: *r*_*s*_ = 0.37). In comparable studies in soccer, Romann et al. (2017) and Malina et al. (2012) found similar correlations (0.26 ≤ *r*_*s*_ ≤ 0.47) between skeletal age and PAH, as well as predicted APHV in 11- to 14-years-old male soccer players. However, these studies mainly used bivariate, correlative approaches to analyze the relationship between the gold standard and further BMS diagnostics.

Therefore, special focus should be given to an accurate and comprehensive investigation of diagnostics' reliability and validity by comparing alternative diagnostics with a well-established reference method (i.e., MRI) from different perspectives. Here, *correlational analyses* (*perspective 1*) give insight into the association between possible appropriate diagnostics and the reference method. Furthermore, a multivariate consideration of the alternative diagnostics' coherence with the *theoretical construct of BMS* (*perspective 2*) may facilitate a more comprehensive view of diagnostics' psychometric properties. Therefore, a structural equation modeling (SEM) approach may be beneficial to define BMS as a latent variable by utilizing the reference method as the measurement model. Consequently, this makes it possible to accurately analyze the degree of the theoretical construct's correspondence with further alternative and more pragmatic diagnostics (e.g., Bollen, 1989). More specifically, one can investigate whether the multiple alternative BMS diagnostics may be utilized in combination to represent BMS in a satisfying manner. Since the consideration of manifest variables as indicators implies neglecting potential measurement errors, SEM could take this problem into account and enable more exact calculations (e.g., Skrondal and Rabe-Hesketh, 2004). However, perspectives 1 and 2 fail to examine absolute differences between two measures (Bland and Altman, 2003). In order to go beyond such an examination of the relationship of the considered diagnostics for criterion validation, the *absolute agreement* (*perspective 3*) between the reference method and the alternative diagnostics should be taken into account by analyzing individual differences and systematic biases in agreement between the various methods (Bland and Altman, 1999; Giavarina, 2015).

## The Present Study

The aim of the present study is to evaluate pragmatic diagnostics for assessing biological maturity in a representative sample of elite youth soccer players by comparing their applicability for the assessment of skeletal age, which is currently considered the gold standard method (Lloyd et al., 2014). In doing so, the reference method MRI was set as the criterion to determine skeletal age (e.g., Serinelli et al., 2015; Urschler et al., 2016). The MRI approach was chosen in order to avoid possible health risks for players due to unnecessary radiation exposure (i.e., X-ray method). In addition to fulfilling economic, ethical, and pragmatic criteria, the study focuses especially on the criterion validation of different diagnostics to assess BMS. Therefore, the study's main purpose was to investigate the agreement between the radiation-free MRI diagnostics and the alternative (e.g., in terms of setup and cost), more economical and practical methods of measuring BMS by

(a) Skeletal age using a quantitative ultrasound-based device and
(b) Somatic age utilizing estimates of MO (Mirwald et al., 2002) and PAH (Khamis and Roche, 1994).

These BMS outcomes were related to the reference method MRI using three perspectives of analyses:

- Bivariate correlation analyses of MRI with the alternative BMS diagnostics,
- Multivariate modeling of BMS as a latent variable (measured by MRI) based on alternative BMS diagnostics, and
- Investigation of individual differences and systematic bias in agreement between MRI and alternative BMS diagnostics.

## Methods

### Participants

The study sample consisted of male youth soccer players (*N* = 63) who were part of the German talent promotion program. Players were born between 2006 and 2008 and belonged to either the U12 (*n* = 32, 11.3 ± 0.3 years old) or U14 age group (*n* = 31, 13.4 ± 0.3 years old). For the estimation of an appropriate sample size in each age group, statistical a priori power analyses were performed utilizing G^*^Power Version 3.1.9.4 (α = 0.05, 1 – β = 0.85, two-tailed). In order to detect at least large effect sizes within the correlational analyses (i.e., *r* ≥ 0.50; Cohen, 1988), a sample size of at least 30 players in each age group (i.e., U12 and U14) was indicated.

As the talent promotion program comprises two important levels of promotion in early to middle adolescence (i.e., competence centers and youth academies), the sample included a balanced amount of competence center (U12: *n* = 16; U14: *n* = 16) as well as youth academy players (U12: *n* = 16; U14: *n* = 15). All players' legal guardian/next of kin provided informed written consent for the collection and scientific use of the data. With respect to the MRI diagnostics, players and their parents were informed about the examination in advance and had to sign a study participation agreement. The research was approved by the ethics committee of the Faculty of Medicine at the University of Frankfurt and the scientific board of the DFB Academy.

### Measures

The entire investigation was conducted within 2 weeks at Frankfurt University Hospital and was predetermined in a strict protocol. Testing for one player, including MRI, ultrasound, and anthropometric data, took about 25 min and took place between 12 and 4 p.m. at the day of assessment. Before every measurement, all players were informed about the detailed assessment procedure by the respective investigators.

### Criterion

To assess the reference method for BMS, an MRI of each player's left hand was taken. A 3.0-Tesla MRI (MAGNETOM Prisma, Siemens, Erlangen, Germany) using a dedicated wrist coil was implemented for the native MRI examination. Players were examined in the prone position with the left arm extended (super-man position). In the coil, the middle finger was positioned in the same axis as the radius to avoid ulnar or dorsal deviation. The MRI data of the bones of the left hand were evaluated by three certified clinical radiologists with different experience levels (1 = specific pediatric radiologist, 2 = more than 20 years, and 3 = more than 3 years of experience in clinical radiology) independently from each other. The conventional Tanner–Whitehouse 2 method (TW2; Tanner et al., 2001; Satoh, 2015) was used to determine the skeletal age to the nearest 0.1 years. Inter-rater reliabilities were found to be excellent for the total sample (*ICC* = 0.988, 95% CI = [0.980; 0.992]) as well as for each age group separately (U12: *ICC* = 0.978, 95% CI = [0.958; 0.989]; U14: *ICC* = 0.979, 95% CI = [0.961; 0.989]). The average of all three raters served as players' skeletal age according to MRI (SA_MRI_).

### Predictors

To determine the *skeletal age* based on the ultrasound examination, the BAUSport™ instrument was used (Rachmiel et al., 2017). The ultrasound device is a small, portable, bone sonometer (SonicBone, Rishon Lezion, Israel). It analyzes three sites of the left hand [(1) the distal radius and ulna's secondary ossification centers of the epiphyses at the wrist; (2) the growth plate of the third metacarpal and the shaft of the proximal phalange; and (3) the distal metacarpal epiphysis at the metacarpals]. The device measures the speed of propagation through bone of inaudible high-frequency waves of a short ultrasound pulse (m/s) and the distance attenuation factor (decay rate). With the use of these parameters, skeletal age was calculated (to the nearest 0.01 years) by an algorithm integrated into the software of BAUSport™ using the scoring method designed by Tanner and Whitehouse (TW2 method; Tanner et al., 2001; Rachmiel et al., 2017). All ultrasound examinations were conducted by a trained person according to the BAUSport™ user manual's instructions. All subjects underwent two measurements. Correlation analyses showed excellent retest reliability for the two measurements (*r*_*tt*_ = 0.98). The mean of both measurements comprised players' skeletal age according to ultrasound (SA_US_).

For anthropometric data assessment, all players were barefoot and wore only shorts. Weight was measured with calibrated scales (seca 213 portable stadiometer) to the nearest 0.1 kg. Height and sitting height were determined to the nearest 0.1 cm with a fixed stadiometer (seca 813 electronic flat scale). Here, players had to stand with feet together and arms relaxed. For sitting height, the players sat on a table with an upright trunk and back against the stadiometer. Leg length was indirectly calculated as the difference between standing height and sitting height. In both measurements, the players' head was aligned with the Frankfurt horizontal plane (Malina and Koziel, 2014). Two measurements were taken for each anthropometric variable by the same trained research assistant. Retest reliabilities for all anthropometric measurements were excellent (*r*_*tt*_ ≥ 0.99). If the results differed by more than 0.4 kg for weight, or 0.4 cm for height, or 0.4 cm for sitting height, a third measurement was conducted (Mirwald et al., 2002). The findings for each anthropometric measurement were averaged.

In order to determine the BMS by *somatic age*, two well-known methods utilizing these anthropometric data were applied. First, players' MO from their PHV (MO_MIR_) was computed based on Mirwald's equation (Mirwald et al., 2002):

MO_MIR_ (in years) = −9.236 + [0.0002708 × (leg length × sitting height)] + [−0.001663 × (CA × leg length)] + [0.007216 × (CA × sitting height)] + [0.02292 × (weight by height ratio ×100)], where the leg length was estimated by subtracting sitting height from height.

By additionally recording the body sizes of the biological parents (collected by a questionnaire), the somatic age was also estimated using the Khamis–Roche method (Khamis and Roche, 1994). The method enables prediction of players' adult height based on the regression formula:

predicted adult height (in cm) = β_0_ + β_1_ × height + β_2_ × weight + β_3_ × mid-parent height,

where β_0_, β_1_, β_2_, and β_3_ represent age and gender-specific regression coefficients defined by Khamis and Roche (1994) (for more details, see this original research). In order to control for a potential overestimation of the self-reported heights by parents (Maukonen et al., 2018) and in line with former research (Cumming et al., 2018b), parents' heights were adjusted according to the recommendations of Epstein et al. (1995) before calculating the mid-parent height parameter. By utilizing this adult height prediction, players' PAH (in %) (PAH_KR_) was calculated by the ratio (height/predicted adult height).

### Data Analysis

Data were analyzed utilizing IBM SPSS version 26 and Mplus Version 8.4. In order to compare the reference method (SA_MRI_) and the alternative diagnostics for assessing BMS (SA_US_, MO_MIR_, and PAH_KR_) according to the three perspectives of analyses, the following statistical procedures were applied.

#### Bivariate Correlation Analyses

Pearson's *r* served as the measure for the correlations between SA_MRI_ and the alternative BMS diagnostics (SA_US_, MO_MIR_, and PAH_KR_) for the total sample as well as for each age group separately.

#### Multivariate Latent Structural Equation Modeling

A SEM approach was used to model BMS as a latent construct. Within the measurement model, three different evaluations of SA_MRI_ by the independent clinical experts were defined to load on the latent variable BMS_lat_. The alternative diagnostics SA_US_, MO_MIR_, and PAH_KR_ served as predictors for BMS_lat_. In accordance with Muthén and Muthén (2010), *R*^2^ was examined to quantify the amount of variance within BMS_lat_ explained by the utilized predictors within the latent regression model. As the sample sizes within each age group were too low to specify a model for U12 and U14 separately, the SEM was only computed for the total sample. However, in order to also adjust for the classification to an age group for this perspective, all variables were z-standardized within each age group before the model was run.

#### Investigation of Individual Differences and Systematic Bias in Agreement

In addition to the correlative approaches in perspectives 1 and 2, Bland–Altman analyses (Bland and Altman, 1999) were utilized to investigate individual differences as well as systematic biases in agreement between SA_MRI_ and each of the three alternative measures SA_US_, MO_MIR_, and PAH_KR_. Since a comparison between two methods is only reasonable when two measurements are of the same unit, some measurements had to be converted before the analysis. In particular, MO_MIR_ was converted into skeletal age (i.e., SA_MIR_) based on the mean individual APHV for boys (i.e., 13.8 years; Malina et al., 2004) via the equation SA_MIR_ = MO_MIR_ + 13.8. With respect to PAH_KR_, there was no possibility for a transformation into skeletal age. For this reason, SA_MRI_ was transformed into values of achieved percentage of adult height (i.e., PAH_MRI_) by a conversion tool BoneXpert, 2020 validated by Thodberg et al. (2009). Finally, players' PAH_MRI_ was determined as the ratio of their current height and their predicted adult height. As the BoneXpert conversion is restricted to individuals where the absolute difference between their skeletal and CA is <3.5 years, three players had to be excluded from this part of the analyses.

In accordance with Bland and Altman (1999), the average of two measures to be compared (i.e., SA_MRI_ and SA_US_, SA_MRI_ and SA_MIR_, and PAH_MRI_ and PAH_KR_, resp.) constituted the x-axis, whereas the differences between the measures (SA_MRI_ – SA_US_, SA_MRI_ – SA_MIR_, and PAH_MRI_ – PAH_KR_, resp.) were depicted on the y-axis of the plots. Additionally, the mean difference and the corresponding 95% limits of agreement were computed and marked within the graphs according to Bland and Altman (2003). Finally, one-sample *t*-tests were utilized in order to examine whether there was a significant systematic bias between two measurements, which was indicated if the average of the differences between measurements deviated significantly from zero.

## Results

Descriptive statistics for all maturity-related outcomes for the total sample as well as separated by age group are displayed in Table 1.

**Table 1:** Descriptive overview of BMS diagnostics' outcomes.

		**Age group**		
**Outcome**		**U12 (*n* = 32)**	**U14 (*n* = 31)**	**Total (*n* = 63)**
			***M*** **±*****SD***	
Anthropometry	Height (cm)	150.06 ± 5.48	164.86 ± 10.23	157.35 ± 11.01
	Weight (kg)	39.13 ± 4.33	51.37 ± 8.88	45.15 ± 9.25
Chronological age	CA (years)	11.33 ± 0.28	13.41 ± 0.29	12.35 ± 1.09
Skeletal age	SA_MRI_ (years)	12.06 ± 0.88	13.86 ± 1.17	12.95 ± 1.37
	SA_US_ (years)	11.75 ± 0.89	14.06 ± 1.44	12.89 ± 1.66
Somatic age	MO_MIR_ (years)	−2.11 ± 0.37	−0.22 ± 0.79	−1.18 ± 1.13
	PAH_KR_ (%)	83.40 ± 1.78	91.64 ± 2.82	87.45 ± 4.76

*SA_MRI_, skeletal age determined by magnetic resonance imaging; SA_US_, skeletal age determined by mobile ultrasound device; MO_MIR_, maturity offset according to Mirwald et al. (2002); PAH_KR_, percentage of adult height according to Khamis and Roche (1994)*.

### Bivariate Correlation Analyses

The results of the correlation analyses with respect to various BMS outcomes are presented in Table 2. All correlations were found to be significant (*p* <0.001). With respect to the total sample, correlation coefficients of SA_MRI_ and the further BMS variables are ranked from *r* = 0.80 (SA_MRI_, SA_US_) to *r* = 0.84 (SA_MRI_, MO_MIR_). When looking at the age groups U12 and U14 separately, correlations for U14 were higher for BMS variables (0.61 ≤ *r* ≤ 0.74) than those for U12 (0.56 ≤ *r* ≤ 0.67).

**Table 2:** Correlation analyses between SA_MRI_ and alternative diagnostics for the total sample and each age class separately.

		**Pearson's** ***r***		
	**Method**	**SA_**US**_**	**MO_**MIR**_**	**PAH_**KR**_**
U12 (*n* = 32)		0.56^***^	0.63^***^	0.66^***^
U14 (*n* = 31)	**SA**_**MRI**_	0.65^***^	0.74^***^	0.61^***^
Total (*n* = 63)		0.80^***^	0.84^***^	0.81^***^

*** *p <0.001*.

*SA_MRI_, skeletal age determined by magnetic resonance imaging; SA_US_, skeletal age determined by mobile ultrasound device; MO_MIR_, maturity offset according to Mirwald et al. (2002); PAH_KR_, percentage of adult height according to Khamis and Roche (1994)*.

### Multivariate Latent Structural Equation Modeling

The analysis of the SEM for the total sample indicated excellent model fit [$\chi_{\left(6\right)}^{2}$ = 1.98, *p* = 0.92, root mean square error of approximation (RMSEA) (90% CI) = 0.00 ([0.00; 0.06]), comparative fit index (CFI) = 1.00, Tucker Lewis index (TFI) = 1.00, standardized root mean square residual (SRMR) = 0.01] and revealed *R*^2^ = 0.51, indicating that 51% of BMS_lat_ variance could be explained by the model.

**Figure 1:**
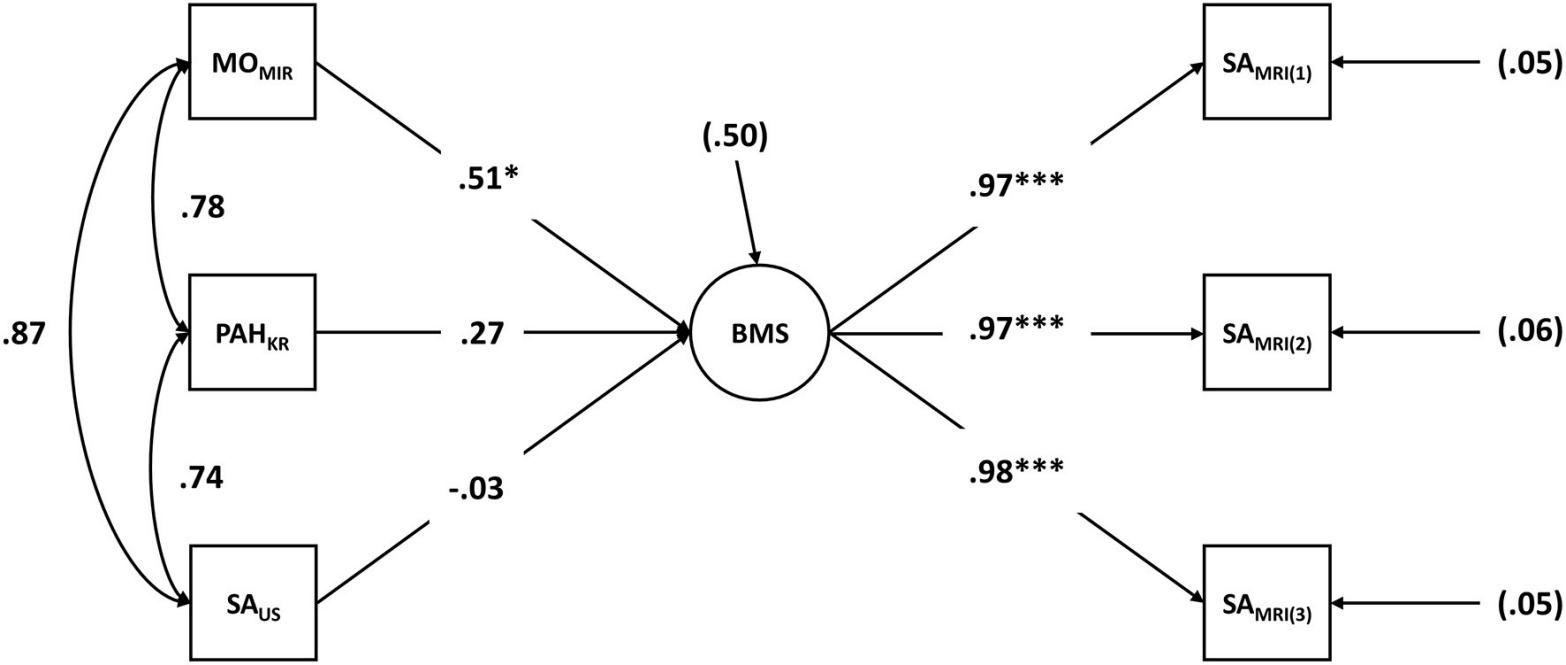
Latent structural equation modeling (SEM): biological maturity status (BMS) as a latent construct predicted by the alternative BMS diagnostics. BMS_lat_, biological maturity status as a latent construct; SA_MRI(i)_, evaluation of skeletal age based on magnetic resonance imaging by rater *i*; SA_US_, skeletal age determined by mobile ultrasound device; MO_MIR_, maturity offset according to Mirwald et al. (2002); PAH_KR_, percentage of adult height according to Khamis and Roche (1994).

Figure 1 presents the estimated model including factor loadings and standardized regression coefficients. First, the manifest MRI evaluations of skeletal age [SA_MRI(1)_, SA_MRI(2)_, and SA_MRI(3)_] loaded particularly highly on BMS_lat_ (0.97 ≤ λ ≤ 0.98; *p* <0.001).

The latent SEM revealed a significant influence on the latent factor BMS_lat_ for the variable MO_MIR_ (β = 0.51, *p* <0.05). Due to high correlations between MO_MIR_ and the other alternative diagnostics PAH_KR_ (*r* = 0.78) and SA_US_ (*r* = 0.87), the additional contribution of PAH_KR_ (β = 0.27, *p* = 0.06), and in particular of SA_US_ (β = −0.03, *p* = 0.90) within the regression model was rather small and not significant.

### Investigation of Individual Differences and Systematic Biases in Agreement

**Figure 2:**
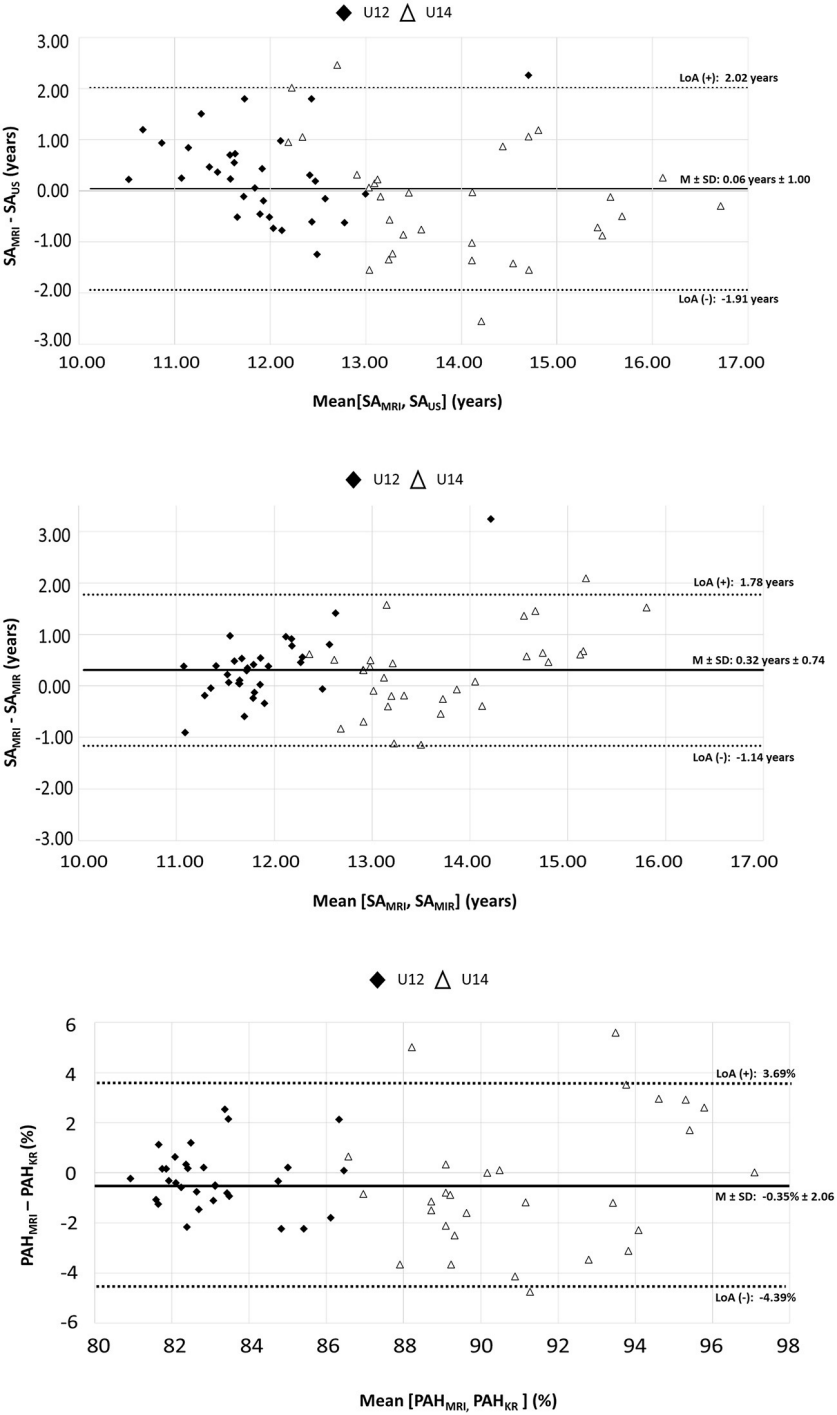
Bland–Altman plots: individual differences of SA_MRI_ and the alternative BMS diagnostics. SA_MRI_, skeletal age determined by magnetic resonance imaging; SA_US_, skeletal age determined by mobile ultrasound device; SA_MIR_, maturity offset (Mirwald et al., 2002) transformed to skeletal age according to Thodberg et al. (2009); PAH_KR_, percentage of adult height according to Khamis and Roche (1994); PAH_MRI_, skeletal age determined by magnetic resonance imaging converted to percentage of adult height according to Thodberg et al. (2009).

The results from the Bland–Altman analyses are shown in Figure 2. When regarding the range of differences between SA_MRI_ and the other diagnostics with increasing mean values between two measurements, differences did not seem to correspond with the mean value. Furthermore, the investigation at an individual level showed that nearly all differences between the reference method (SA_MRI_) and each alternative BMS diagnostics were within the 95% limits of agreement for the mean value. In total, five individuals were identified as outliers (i.e., players whose differences fell outside of the 95% limits of agreement) by at least one of the three comparisons between the reference method and the pragmatic BMS diagnostics. All three outliers identified by SA_US_ were also detected by at least one further comparison. However, both SA_MIR_ and PAH_KR_ found one outlier each that was not recognized by another comparison. Moreover, while the average of the differences for the comparison of SA_MRI_ and the SA_MIR_ deviated significantly from zero [*M* = 0.32 years, *t*_(62)_ = 3.45, *p* <0.01], no systematic bias was found for the comparisons of SA_MRI_ with SA_US_ [*M* = 0.06 years, *t*_(62)_ = 0.45, *p* = 0.65] as well as with PAH_KR_ [*M* = −0.35%, *t*_(59)_ = −1.30, *p* = 0.20]. However, considerable variation with regard to the individual differences was found for all three comparisons (*SD* = 0.74 years for SA_MIR_, *SD* = 2.06% for PAH_KR_, *SD* = 1.00 years for SA_US_, respectively).

## Discussion

In addressing the demand of both practitioners and researchers to integrate information about players' BMS within the processes of talent promotion, the present study evaluated various BMS diagnostics within a representative setting. Highly talented adolescent players (i.e., U12 and U14) from the two main institutions of the German talent promotion program (i.e., competence centers and youth academies) underwent a test battery consisting of the (costly and time intensive) reference method (SA_MRI_) as well as additional, more pragmatic diagnostics (SA_US_, MO_MIR_, and PAH_KR_) that could be applied, among other things, in an area-wide setting. Following the idea of a comprehensive evaluation, diagnostics were related to the reference method from three different perspectives (i.e., bivariate correlation analyses, multivariate latent SEM approach, and investigation of individual differences and systematic deviations). The comparison between the reference method and the alternative diagnostics (*perspective 1*) revealed strong correlations for the total sample (*r* > 0.80) and, as expected due to a lower variance in age, slightly lower correlations regarding U12 (*r* ≥ 0.56) and U14 (*r* ≥ 0.61) separately. The multivariate SEM approach (*perspective 2*) allowed for an accurate investigation (adjusted for age group) of the alternative diagnostics' conformity with BMS as a latent construct free of measurement errors (BMS_lat_). BMS_lat_ was measured by three evaluations of the reference method by independent experts. Overall, the three alternative BMS outcomes (in combination) predicted significant BMS_lat_. *Perspective 3* added value to the diagnostics' evaluation by considering differences and systematic deviations at the individual level. The procedures' average difference exposed a systematic bias for SA_MIR_. Furthermore, the comparisons revealed high standard deviations for the differences between the reference method and the pragmatic diagnostics. With respect to detected outliers, a high degree of agreement was achieved among comparisons.

Former research that aimed to validate different diagnostics to assess BMS in youth players mainly utilized correlative approaches, that is, *perspective 1* (e.g., Malina et al., 2007b in American football; Malina et al., 2012 and Romann et al., 2017 in soccer; Myburgh et al., 2019 in tennis). While the players in those studies were of similar CA as were participants of the present study, all authors used maturity categories (early, on time, and late) instead of continuous outcomes to define players' BMS. This may limit the comparability to the present study, which utilized continuous BMS variables (e.g., skeletal age) in order to differentiate more precisely between diagnostics. Nevertheless, the results of categorical classifications were compared with the results of the current study to establish a reference to existing literature. In general, the correlations between diagnostics were found to be higher in the present study. In a study of 143 male American football players (9.27–14.24 years), Malina et al. (2007b) found lower correlations (*r*_*s*_ = 0.52) between the skeletal age measured by left hand-wrist radiographs (evaluated by the Fels method) and PAH_KR_ compared with the results of this investigation (*r* = 0.84). Similar (even lower) results were found by Myburgh et al. (2019) in a study with 40 male, British junior tennis players (12.5 ± 1.8 years). Utilizing the same method as Malina et al. (2007b), this study evaluated PAH_KR_ as well as the predicted APHV (Mirwald et al., 2002) and found limited agreement between various assessment methods (PAH_KR_: *r*_*s*_ = 0.35; predicted APHV: *r*_*s*_ = 0.37). With respect to comparable studies in soccer, Romann et al. (2017) found only moderate rank correlations (*r*_*s*_ = 0.42) between skeletal age (measured by X-ray) and predicted APHV in male Swiss soccer players (13.9 ± 1.8 years), while SA_MRI_ and MO_MIR_ highly correlated within this investigation (*r* = 0.81). A similar pattern holds true for a study of 11- to 12-years-old (*n* = 87) and 13- to 14-years-old (*n* = 93) male soccer players evaluating the relationship among indicators (i.e., skeletal age based on the Fels method, predicted APHV, and PAH_KR_) of BMS (Malina et al., 2012). While results showed small to moderate Spearman rank correlations for both age groups (11–12 years:.26 ≤ *r*_*s*_ ≤ 0.43; 13–14 years: 29 ≤ *r*_*s*_ ≤ 0.47), Pearson coefficients of the present study for the corresponding age groups (U12:.56 ≤ *r* ≤ 0.67; U14:.61 ≤ *r* ≤ 0.74) were large. These higher correlations could be explained by the more differentiated assessment of BMS outcomes (i.e., categorized vs. continuous), which might be indicated when evaluating potential alternative BMS diagnostics.

Moreover, the research analyzing multivariate correspondence between diagnostics is scarce. An exception is the study of Malina et al. (2012), which evaluates the relationship among BMS indicators in young soccer players. The authors found that the chosen indicators (i.e., skeletal age based on radiographs, APHV, PAH_KR_, and stage of pubic hair) showed one principal factor (i.e., one dimension) within a principal component analysis for 13- to 14-years-old players. This finding is in line with the results of the present study where the three alternative diagnostics significantly predicted BMS_lat_ (*perspective 2*). This provides evidence that, in a realistic setting of highly selected, male youth soccer players, alternative diagnostics, such as SA_US_, MO_MIR_, and PAH_KR_ may be used to assess BMS more pragmatically and efficiently in order to incorporate players' BMS as an important criterion within the talent promotion process (i.e., in terms of selections and bio-banding; Cumming, 2018). However, it was particularly MO_MIR_ (β = 0.51) that predicted BMS_lat_ within the regression model. The influences of SA_US_ as well as PAH_KR_ were rather small (β ≤ 0.27) because of the high correlations (*r* ≥ 0.78) within the three alternative measurements (i.e., collinearity). Perhaps the use of similar information to compute BMS (i.e., CA and anthropometric measurements) within those measurements, among other factors, may have led to these high correlations. On the one hand, this may lead to the conclusion that the use of only one measurement may be sufficient. On the other hand, the use of the combination of the three alternative methods leads to a higher degree of explained variance without overly magnifying the efforts that come with the assessment.

A further benefit of such a combinatory approach could be obtained from the investigation of individual differences between the various diagnostics providing a more comprehensive view of players' BMS (e.g., to detect systematic bias between two diagnostics). As demonstrated in *perspective 3*, systematic bias was found between the reference method and the measurement SA_MIR_. Although these pragmatic diagnostics may be easily utilized within an area-wide setting (e.g., a huge number of players within a nationwide promotion program), their use at the individual level must be considered with caution. The systematic bias for the comparison with SA_MIR_ as well as the slightly high variance (e.g., *SD* = 0.74 years for SA_MIR_) indicates considerable deviations between the alternative diagnostics and the reference method. These distinct differences seem problematic when using one of the alternative diagnostics in practice in order to get reliable BMS information at an individual level. Instead, the use of at least two alternative diagnostics may be helpful in order to adjust for the deviations between the pragmatic and reference method diagnostics.

Consequently, the findings from the present study may help practitioners aiming to integrate information about players' BMS within talent promotion. *Perspective 1* showed that all considered alternative diagnostics correlate highly with the reference method and, therefore, may be used as more economic assessment methods for BMS. Similarly, *perspective 2* revealed that a combined, multivariate use of the alternative measurements significantly predicted BMS and led to slightly higher explanatory power. Even though MO_MIR_ provided the highest impact on BMS, the strong correlations between the pragmatic diagnostics did not allow the conclusion of which diagnostics should be preferred. In contrast to perspectives 1 and 2, *perspective 3* was able to detect individual differences and systematic deviations that might be controlled for by using more than one pragmatic BMS diagnostics in practice.

### Limitations and Perspectives

While the main focus within the present study was the investigation of BMS, further aspects of the maturation process, namely, “maturity timing” and “maturity tempo,” may be considered to determine the biological maturity of youth players in sports (Malina et al., 2019). The maturity timing approach describes specific maturational events that occur at a certain point of time at a different CA for every player (Swain et al., 2018). Such events include the estimated APHV (te Wierike et al., 2015), menarche status (Lloyd et al., 2014), or the age of first ejaculation (Mattila et al., 2008). In addition to these objective diagnostics, further approaches in talent research exist that determine maturity timing morphologically by holistic, subjective expert judgments (Romann et al., 2017). In those assessments, responsible coaches evaluate players independently according to certain characteristics (e.g., morphology). From an economic perspective, such a method offers advantages; however, a certain level of experience is essential, and comprehensive evaluation of the reliability and validity of these expert judgments is still pending. For both objective and subjective approaches, individuals are categorized in early, on-time, and late maturing players (e.g., Romann et al., 2017; Myburgh et al., 2019). Maturity tempo examines how fast/slow a child develops biologically (Mendle et al., 2019) and refers to the rate at which maturation progresses between (at least) two measurement points (Howard et al., 2016; Malina, 2017; Radnor et al., 2018). As with BMS and maturity timing, various approaches to determine maturity tempo exist in the literature (e.g., rate between beginning and end of the adolescent growth spurt; Wormhoudt et al., 2017).

However, there is disagreement in the literature with regard to the inconsistency of definitions (Cheng et al., 2020) and which indicators are assigned to which approaches (BMS vs. maturity timing vs. maturity tempo). For example, several authors use APHV (Buchheit and Mendez-Villanueva, 2014; Deprez et al., 2015) as an indicator of BMS, despite the fact that the review by Swain et al. (2018) argues that APHV reflects an indicator of maturity timing. To the authors' best knowledge, both approaches are possible but investigate different aspects; a more precise consideration of this issue is needed. While MO_MIR_ should be used as an indicator of BMS (as in the present study), the difference of a player's individual APHV to the general APHV for boys (i.e., 13.8 years; Malina et al., 2004) provides an indicator of maturity timing. For instance, Mirwald's equation (Mirwald et al., 2002) calculates both BMS and maturity timing. Consequently, future research should carefully choose the right approach for determining an indicator that corresponds to the specific aspect of the maturation process to be investigated. While the present study analyzed BMS outcomes, maturity timing and maturity tempo outcomes—ideally in a longitudinal research design—would be of future interest.

As a limitation of the present study and of the pragmatic assessment of indicators of somatic age in general, it has to be considered that both MO_MIR_ and PAH_KR_ appear to be very sensitive for parameters, such as leg length and standing height. Therefore, in order to ensure precise measurement of these parameters, practitioners should—beyond the use of calibrated measurement devices—control for potential physiological confounding variables. For instance, height and weight might vary at different times of the day (e.g., in the morning/evening or before/after practice; Orsama et al., 2014). For this reason, practitioners should try to maintain a standardized measurement procedure by determining consistent time slots for measuring their players. In addition, concerning the PAH_KR_ method including a mid-parent height parameter, it has been remarked that people tend to overestimate their own height (Maukonen et al., 2018). This, in turn, may falsify the PAH_KR_ values for the respective player and indicates the need for an objective assessment by an independent observer. However, research investigating the measurement errors of PAH_KR_ values between self-reported parents' height and objectively assessed parents' height by researchers is scarce. To the authors' best knowledge, only one equation exists in which the self-reported height is adjusted, developed by Epstein et al. (1995). While this equation was used in the present study as well as in some current studies (e.g., Cumming et al., 2018b), more research is needed for finding an accustomed correction formula to reduce measurement errors based on overestimating self-reported height.

Moreover, players' ethnicity status was not taken into account in this study. Researchers controversially discuss a potential influence of ethnicity on skeletal age. While Timme et al. (2017) emphasize that no impact of ethnicity exists, current studies found significant differences in skeletal age between African and European population (e.g., Grgic et al., 2020). However, the focus of the present study lays in the comparison of different pragmatic methods with the MRI diagnostics, not least because of the effort in terms of time and costs associated with the MRI diagnostics, and the study's sample size was too small to examine the impact on different ethnic groups. Thus, comprehensive validation studies are needed to investigate potential differences when determining BMS for several ethnic groups. Therefore, future studies—ideally in a longitudinal design—should control for a possible impact of ethnicity when examining BMS, and the use of an ethnicity-specific formula might be helpful for this issue. However, to date, there is no formula that could account for ethnicity-specific assessment of BMS.

## Conclusion

The results suggest that the use of SA_US_, MO_MIR_, and PAH_KR_ for measuring BMS is more pragmatic in terms of cost and time as compared with MRI diagnostics. Based on a general agreement between these pragmatic diagnostics and the reference method MRI in all three perspectives, the alternative methods can be used to determine BMS among (male) elite youth soccer players. Since caution is required with respect to the precision of the measurements at the individual level, the simultaneous use of at least two alternative diagnostics is recommended in order to get a more reliable BMS outcome. Further research is needed that evaluates both the implementation of BMS' diagnostics in practice and their usefulness in terms of bio-banding in youth soccer (e.g., Romann et al., 2020).

## Data Availability Statement

A de-identified version of the raw data supporting the conclusions of this article the findings of this study will be made available by the authors upon reasonable request.

## Ethics Statement

The studies involving human participants were reviewed and approved by Ethics committee of the Faculty of Medicine at the University of Frankfurt. Written informed consent to participate in this study was provided by the participants' legal guardian/next of kin.

## Author Contributions

OH, DLe, and DM: conceptualization and methodology. OH: data curation and supervision. DLe: formal analysis. OH and TH: funding acquisition. OH, DLe, DM, GS, MR, DLü, LB, and KE: investigation. OH, MR, and KE: project administration. DLe and DM: validation, visualization, and writing ± original draft. DLe, DM, OH, LB, KE, TH, DLü, MR, and GS: writing ± review and editing. All authors contributed to the article and approved the submitted version.

## Conflict of Interest

The authors declare that the research was conducted in the absence of any commercial or financial relationships that could be construed as a potential conflict of interest.

## References

[B1] Baker J. Schorer J. Wattie N. (2018). Compromising talent: issues in identifying and selecting talent in Sport. Quest 70, 48–63. 10.1080/00336297.2017.1333438

[B2] Bland J. Altman D. (1999). Measuring agreement in method comparison studies. Stat. Methods Med. Res. 8, 135–160. 10.1177/096228029900800204 10501650

[B3] Bland J. Altman D. (2003). Applying the right statistics: analyses of measurement studies. Ultrasound Obstetr. Gynecol. 22, 85–93. 10.1002/uog.122 12858311

[B4] Bolívar J. Sandoval Ó. Osorio J. Dib G. Gallo J. (2015). Relationship of chronological age and sexual maturity with skeletal maturity by magnetic resonance imaging of the distal radial epiphysis in adolescent football players. Apunts Sports Med. 50, 129–137. 10.1016/j.apunts.2015.05.002

[B5] Bollen K. (1989). Structural Equations with Latent Variables. New York, NY: John Wiley & Sons.

[B6] BoneXpert (2020). Adult Height Predictor. Retrieved from: https://bonexpert.com/adult-height-predictor/

[B7] Bradley B. Johnson D. Hill M. McGee D. Kana-Ah A. Sharpin C.. (2019). Bio-banding in academy football: player's perceptions of a maturity matched tournament. Ann. Hum. Biol. 46, 400–408. 10.1080/03014460.2019.1640284 31288575

[B8] Buchheit M. Mendez-Villanueva A. (2014). Effects of age, maturity and body dimensions on match running performance in highly trained under-15 soccer players. J. Sports Sci. 32, 1271–1278. 10.1080/02640414.2014.884721 24786981

[B9] Cheng H. Harris S. Sritharan M. Behan M. Medlow S. Steinbeck K. (2020). The tempo of puberty and its relationship to adolescent health and well-being: a systematic review. Acta Paediatr. 109, 900–913. 10.1111/apa.15092 31730292

[B10] Cohen J. (1988). Statistical Power Analysis for the Behavioral Sciences. Hillsdale, NJ: Lawrence Erlbaum Associates.

[B11] Cumming S. (2018). A game plan for growth: how football is leading the way in the consideration of biological maturation in young male athletes. Ann. Hum. Biol. 45, 373–375. 10.1080/03014460.2018.1513560 30767617

[B12] Cumming S. Brown D. J. Mitchell S. Bunce J. Hunt D. Hedges C.. (2018a). Premier League academy soccer players' experiences of competing in a tournament bio-banded for biological maturation. J. Sports Sci. 36, 757–765. 10.1080/02640414.2017.1340656 28628369

[B13] Cumming S. Lloyd R. S. Oliver J. L. Eisenmann J. Malina R. (2017). Bio-banding in sport: applications to competition, talent identification, and strength and conditioning of youth athletes. Strength Cond. J. 39, 34–47. 10.1519/SSC.0000000000000281

[B14] Cumming S. Searle C. Hemsley J. Haswell F. Edwards H. Scott S. (2018b). Biological maturation, relative age and self-regulation in male professional academy soccer players: a test of the underdog hypothesis. Psychol. Sport Exerc. 39, 147–153. 10.1016/j.psychsport.2018.08.007

[B15] Deprez D. Coutts A. Fransen J. Deconinck F. Lenoir M. Vaeyens R.. (2013). Relative age, biological maturation and anaerobic characteristics in elite youth soccer players. Int. J. Sports Med. 34, 897–903. 10.1055/s-0032-1333262 23700327

[B16] Deprez D. Buchheit M. Fransen J. Pion J. Lenoir M. Philippaerts R.. (2015). A longitudinal study investigating the stability of anthropometry and soccer-specific endurance in pubertal high-level youth soccer players. J. Sports Sci. Med. 14, 418–426. 25983593 PMC4424473

[B17] Deutscher Fußball-Bund (2020). DFB Jugendordnung [DFB Youth Regulations]. Retrieved from: https://www.dfb.de/fileadmin/_dfbdam/218062-11_Jugendordnung.pdf

[B18] Dvorak J. (2009). Detecting over-age players using wrist MRI: science partnering with sport to ensure fair play. Br. J. Sport. Med. 43, 884–885. 10.1136/bjsm.2009.067439 19843559

[B19] Dvorak J. George J. Junge A. Hodler J. (2007). Age determination by magnetic resonance imaging of the wrist in adolescent male football players. Br. J. Sports Med. 41, 45–52. 10.1136/bjsm.2006.031021 17021001 PMC2465138

[B20] Epstein L. H. Valoski A. M. Kalarchian M. A. McCurley J. (1995). Do children lose and maintain weight easier than adults: a comparison of child and parent weight changes from six months to ten years. Obes. Res. 3, 411–417. 10.1002/j.1550-8528.1995.tb00170.x 8521160

[B21] Focardi M. Pinchi V. de Luca F. Norelli G. (2014). Age estimation for forensic purposes in Italy: ethical issues. Int. J. Legal Med. 128, 515–522. 10.1007/s00414-014-0986-0 24633466

[B22] Fraser-Thomas J. Côté J. Deakin J. (2008). Understanding dropout and prolonged engagement in adolescent competitive sport. Psychol. Sport Exerc. 9, 645–662. 10.1016/j.psychsport.2007.08.003

[B23] George J. Nagendran J. Azmi K. (2012). Comparison study of growth plate fusion using MRI versus plain radiographs as used in age determination for exclusion of overaged football players. Br. J. Sports Med. 46:273. 10.1136/bjsm.2010.074948 21173009

[B24] Giavarina D. (2015). Understanding bland Altman analysis. Biochem. Med. 25, 141–151. 10.11613/BM.2015.015 26110027 PMC4470095

[B25] Gouvêa M. Cyrino E. Valente-Dos-Santos J. Ribeiro A. Silva D. Ohara D. (2017). Comparison of skillful vs. less skilled young soccer players on anthropometric, maturation, physical fitness and time of practice. Int. J. Sports Med. 38:384–395. 10.1055/s-0042-122815 28340491

[B26] Grgic O. Shevroja E. Dhamo B. Uitterlinden A. G. Wolvius E. P. Rivadeneira F.. (2020). Skeletal maturation in relation to ethnic background in children of school age: the Generation R Study. Bone 132:115180. 10.1016/j.bone.2019.115180 31786375

[B27] Helsen W. van Winckel J. Williams A. (2005). The relative age effect in youth soccer across Europe. J. Sports Sci. 23, 629–636. 10.1080/02640410400021310 16195011

[B28] Holienka M. Babic M. DoleŽajová L. Šelinger P. Musilová E. (2017). Motor performance of young soccer players based on their biological age. J. Phys. Educ. Sport 17, 2508–2512. 10.7752/jpes.2017.04282

[B29] Howard S. Cumming S. Atkinson M. Malina R. (2016). Biological maturity-associated variance in peak power output and momentum in academy rugby union players. Eur. J. Sport Sci. 16, 972–980. 10.1080/17461391.2016.1205144 27485020

[B30] Johnson A. Farooq A. Whiteley R. (2017). Skeletal maturation status is more strongly associated with academy selection than birth quarter. Sci. Med. Football 1, 157–163. 10.1080/24733938.2017.1283434

[B31] Johnston K. Wattie N. Schorer J. Baker J. (2018). Talent identification in sport: a systematic review. Sport Med. 48, 97–109. 10.1007/s40279-017-0803-2 29082463

[B32] Khamis H. Roche A. (1994). Predicting adult stature without using skeletal age: the Khamis-Roche method. Pediatrics 94, 504–507. 7936860

[B33] Lloyd R. Oliver J. Faigenbaum A. Myer G. De Ste Croix M. (2014). Chronological age vs. biological maturation: implications for exercise programming in youth. J. Strength Cond. Res. 28, 1454–1464. 10.1519/JSC.0000000000000391 24476778

[B34] Malina R. (2017). “Assessment of biological maturation,” in Oxford Textbook of Children's Exercise Science and Medicine, eds N. Armstrong and W. Mechelen (Oxford: University Press), 3–11. 10.1093/med/9780198757672.003.0001

[B35] Malina R. Bouchard C. Bar-Or O. (2004). Growth, Maturation, and Physical Activity. Champaign, IL: Human Kinetics Publihsers, Inc.

[B36] Malina R. Chamorro M. Serratosa L. Morate F. (2007a). TW3 and Fels skeletal ages in elite youth soccer players. Ann. Hum. Biol. 34, 265–272. 10.1080/03014460701207601 17558596

[B37] Malina R. Coelho-e-Silva M. Figueiredo A. Carling C. Beunen G. (2012). Interrelationships among invasive and non-invasive indicators of biological maturation in adolescent male soccer players. J. Sports Sci. 30, 1705–1717. 10.1080/02640414.2011.639382 22304621

[B38] Malina R. Cumming S. Rogol A. D. Coelho-e-Silva M. Figueiredo A. Konarski J.. (2019). Bio-banding in youth sports: background, concept, and application. Sports Med. 49, 1671–1685. 10.1007/s40279-019-01166-x 31429034

[B39] Malina R. Dompier T. Powell J. Barron M. Moore M. (2007b). Validation of a noninvasive maturity estimate relative to skeletal age in youth football players. Clin. J. Sports Med. 17, 362–368. 10.1097/JSM.0b013e31815400f4 17873548

[B40] Malina R. Koziel S. (2014). Validation of maturity offset in a longitudinal sample of polish girls. J. Sports Sci. 32, 1374–1382. 10.1080/02640414.2014.889846 24892233

[B41] Malina R. Rogol A. Cumming S. Coelho e Silva M. Figueiredo A. (2015). Biological maturation of youth athletes: assessment and implications. Br. J. Sports Med. 49, 852–859. 10.1136/bjsports-2015-094623 26084525

[B42] Mattila V. M. Saarni L. Parkkari J. Koivusilta L. Rimpelä A. (2008). Predictors of low back pain hospitalization–a prospective follow-up of 57,408 adolescents. Pain 139, 209–217. 10.1016/j.pain.2008.03.028 18472217

[B43] Maukonen M. Männistö S. Tolonen H. (2018). A comparison of measured versus self-reported anthropometrics for assessing obesity in adults: a literature review. Scand. J. Public Health 46, 565–579. 10.1177/1403494818761971 29528773

[B44] Mendle J. Beltz A. Carter R. Dorn L. (2019). Understanding puberty and its measurement: ideas for research in a new generation. J. Res. Adolesc. 29, 82–95. 10.1111/jora.12371 30869839

[B45] Mirwald R. Baxter-Jones A. Bailey D. Beunen G. (2002). An assessment of maturity from anthropometric measurements. Med. Sci. Sports Exerc. 34, 689–694. 10.1249/00005768-200204000-00020 11932580

[B46] Murr D. Raabe J. Höner O. (2018). The prognostic value of physiological and physical characteristics in youth soccer: a systematic review. Eur. J. Sport Sci. 18, 62–74. 10.1080/17461391.2017.1386719 29161984

[B47] Muthén L. Muthén B. (2010). Mplus: Statistical Analysis With Latent Variables; Users Guide, 6th Edn. Los Angeles, CA: Muthén & Muthén.

[B48] Myburgh G. Cumming S. Malina R. (2019). Cross-sectional analysis investigating the concordance of maturity status classifications in elite caucasian youth tennis players. Sport. Med. Open 5:27. 10.1186/s40798-019-0198-8 31264052 PMC6603099

[B49] Orsama A. L. Mattila E. Ermes M. van Gils M. Wansink B. Korhonen I.. (2014). Weight rhythms: weight increases during weekends and decreases during weekdays. Obes. Facts. 7, 36–47. 10.1159/000356147 24504358 PMC5644907

[B50] Rachmiel M. Naugolni L. Mazor-Aronovitch K. Koren-Morag N. Bistritzer T. (2017). Bone age assessments by quantitative ultrasound (sonicbone) and hand x-ray based methods are comparable. Isr. Med. Assoc. J. 19, 533–538. 28971634

[B51] Radnor J. M. Oliver J. L. Waugh C. M. Myer G. D. Moore I. S. Lloyd R. S. (2018). The influence of growth and maturation on stretch-shortening cycle function in youth. Sport. Med. 48, 57–71. 10.1007/s40279-017-0785-0 28900862 PMC5752749

[B52] Romann M. Lüdin D. Born D. (2020). Bio-banding in junior soccer players: a pilot study. BMC Res. Notes 13:240. 10.1186/s13104-020-05083-5 32398110 PMC7216411

[B53] Romann M. Javet M. Fuchslocher J. (2017). Coache's eye as a valid method to assess biological maturation in youth elite soccer. Talent Dev. Excell 9, 3–13. Available online at: http://www.iratde.com/index.php/jtde/article/view/1

[B54] Romann M. Javet M. (2018). Massnahmen zur Reduzierung von Age Effects. Magglingen: Bundesamt für Sport BASPO.

[B55] Satoh M. (2015). Bone age: assessment methods and clinical applications. Clin. Pediatr. Endocrinol. 24, 143–152. 10.1297/cpe.24.143 26568655 PMC4628949

[B56] Serinelli S. Panebianco V. Martino M. Battisti S. Rodacki K. Marinelli E.. (2015). Accuracy of MRI skeletal age estimation for subjects 12–19. Potential use for subjects of unknown age. Int. J. Legal Med. 129, 609–617. 10.1007/s00414-015-1161-y 25721414

[B57] Skrondal A. Rabe-Hesketh S. (2004). Generalized Latent Variable Modelling: Multilevel, Longitudinal, and Structural Equation Models. Boca Raton, FL: CRC; Chapman & Hall.

[B58] Swain M. Kamper S. Maher C. Broderick C. McKay D. Henschke N. (2018). Relationship between growth, maturation and musculoskeletal conditions in adolescents: a systematic review. Br. J. Sport. Med. 52, 1246–1252. 10.1136/bjsports-2017-098418 29559438

[B59] Tanner J. Whitehouse R. Cameron N. Marshall W. Healy M. Goldstein H. (2001). Assessment of Skeletal Maturity and Prediction of Adult Height (TW3 Method). London: Saunders.

[B60] te Wierike S. Elferink-Gemser M. Tromp E. Vaeyens R. Visscher C. (2015). Role of maturity timing in selection procedures and in the specialisation of playing positions in youth basketball. J. Sports Sci. 33, 337–345. 10.1080/02640414.2014.942684 25083799

[B61] Thodberg H. Jenni O. Caflisch J. Ranke M. Martin D. (2009). Prediction of adult height based on automated determination of bone age. J. Clin. Endocrinol. Metab. 94, 4868–4874. 10.1210/jc.2009-1429 19926715

[B62] Timme M. Steinacker J. Schmeling A. (2017). Age estimation in competitive sports. Int. J. Legal Med. 131, 225–233. 10.1007/s00414-016-1456-7 27743021

[B63] Unnithan V. White J. Georgiou A. Iga J. Drust B. (2012). Talent identification in youth soccer. J. Sports Sci. 30, 1719–1726. 10.1080/02640414.2012.731515 23046427

[B64] Urschler M. Krauskopf A. Widek T. Sorantin E. Ehammer T. Borkenstein M.. (2016). Applicability of Greulich–Pyle and Tanner–Whitehouse grading methods to MRI when assessing hand bone age in forensic age estimation: a pilot study. Forensic Sci. Int. 266, 281–288. 10.1016/j.forsciint.2016.06.016 27344264

[B65] Utczas K. Muzsnai A. Cameron N. Zsakai A. Bodzsar E. (2017). A comparison of skeletal maturity assessed by radiological and ultrasonic methods. Am. J. Hum. Biol. 29:e22966. 10.1002/ajhb.22966 28094893

[B66] Votteler A. Höner O. (2017). Cross-sectional and longitudinal analyses of the relative age effect in German youth football. German J. Exerc. Sport. Res. 47, 194–204. 10.1007/s12662-017-0457-0

[B67] Wormhoudt R. Savelsbergh G. J. Teunissen J. W. Davids K. (2017). The Athletic Skills Model: Optimizing Talent Development Through Movement Education. London: Routledge.

